# Supplementary material for: The cognitive effects of a promised bonus do not depend on dopamine synthesis capacity
Source: Sci Rep. 2020 Oct 5;10:16473. doi: 10.1038/s41598-020-72329-4 (PMC7536197; doi:10.1038/s41598-020-72329-4)
Supplement: Supplementary file 1 [file 41598_2020_72329_MOESM1_ESM.docx]

**SUPPLEMENTARY INFORMATION**

The cognitive effects of a promised bonus do not depend on dopamine synthesis capacity

**AUTHORS**

Lieke Hofmans*^1,2^, Ruben van den Bosch^1,2^, Jessica I. Määttä^1,2^, Robbert-Jan Verkes^2,3,4^, Esther Aarts^1^, Roshan Cools^1,2^

**AFFILIATIONS**

^1^Donders Institute for Brain, Cognition & Behaviour, Radboud University, Nijmegen, The Netherlands ^2^Department of Psychiatry, Radboudumc, Nijmegen, The Netherlands

^3^Forensic Psychiatric Centre Nijmegen, Pompestichting, Nijmegen, The Netherlands.

^4^Department of Criminal Law, Law School, Radboud Universiteit, Nijmegen, The Netherlands

*** CORRESPONDING AUTHOR**

Lieke Hofmans, Kapittelweg 29 r.2.269, 6525EN Nijmegen, The Netherlands

tel.: +310243614305 | Fax: +310243610989

e-mail: l.hofmans@donders.ru.nl

**Comparison between the regions of interest used in the current study and the study by Aarts *et al.***

Here we report results from a comparison between the regions of interest (ROIs) used in the current study and those used in the study by Aarts and colleagues ^1^. In the current study we based our ROIs on functional connectivity analyses ^2^ (see PET analysis in main text), while the ROIs in the study by Aarts and colleagues were drawn according to guidelines described previously by Mawlawi and colleagues ^3^.

The ROIs as used by Aarts et al. were specified in MNI space and transformed to subject native space for analyses. Figure S1 displays the two sets of ROIs in MNI space. Both sets of ROIs included the bilateral caudate nucleus (medial caudate region and dorsal caudate nucleus region), putamen (dorsal-anterior and dorsal-posterior putamen region) and ventral striatum (nucleus accumbens, ventral caudate nucleus, and ventral parts of the putamen). A Pearson’s correlations revealed that there was a strong positive correlation between the [^18^F]DOPA K_i_ values in the two sets of ROIs (native space; left caudate nucleus: *r* = 0.97, *p* < 2e^-16^; right caudate nucleus: *r* = 0.96, *p* < 2e^-16^; left putamen: *r* = 0.99, *p* < 2e^-16^; right putamen: *r* = 0.99, *p* < 2e^-16^; left ventral striatum: *r* = 0.98, *p* < 2e^-16^; right ventral striatum: *r* = 0.97, *p* < 2e^-16^).

Next we reran the rmANOVAs on the dependent variable Stroop interference – mean RT incongruent trials minus mean RT congruent trials – including the within-subjects factors REWARD (low, high) and INFORMATION (uninformed, informed), and [^18^F]DOPA K_i_ in the left dorsal caudate nucleus (as specified according to Mawlawi *et al.* and used by Aarts *et al.*) as a covariate of interest. There were no significant interactions between REWARD, INFORMATION and dopamine synthesis capacity (*F*_(1,42)_ = 2.2, *p* = 0.150, BF_INC_ = 0.003) or between REWARD and dopamine synthesis capacity (independent of INFORMATION; *F*_(1,42)_ = 0.1, *p* = 0.715, BF_INC_ = 0.044). Figure S2 shows the correlation between dopamine synthesis capacity in the left dorsal caudate nucleus (as specified according to Mawlawi *et al.*) and the effect of motivation on Stroop interference on uninformed trials.


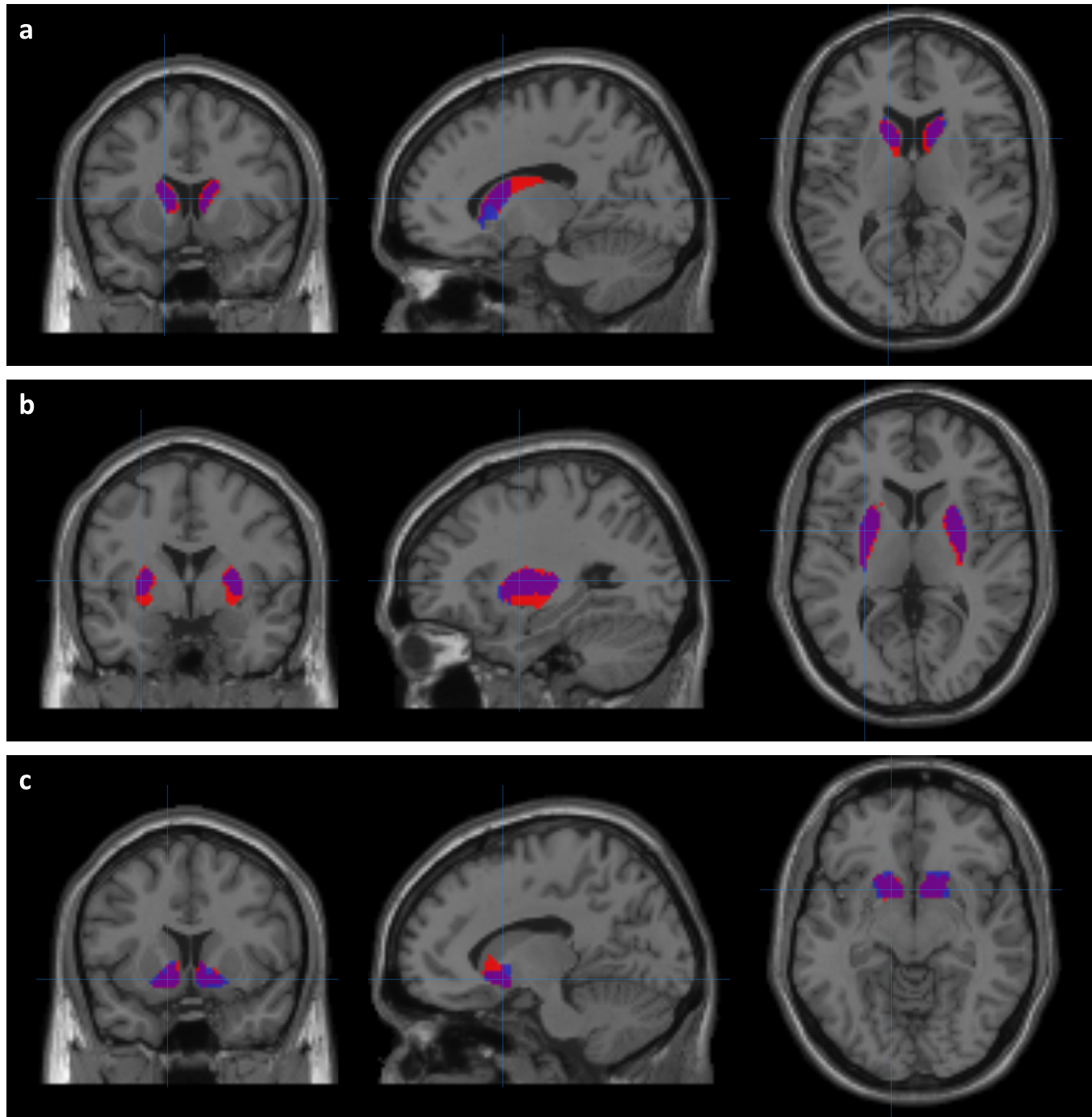
**Figure S1.** Overlay of the regions of interest (ROIs) used in the current study and in Aarts *et al*. ^1^. ROIs used in the current study are displayed in red; ROIs used in Aarts *et al.* are displayed in blue; overlap is purple. **a** – caudate nucleus with crosshairs at MNI coordinates [-14, 10, 10]. **b** – putamen with crosshairs at MNI coordinates [-28, 0, 6]. **c** – ventral striatum with crosshairs at MNI coordinates [-12, 10, -8].

**Figure S2.** The effect of reward on Stroop interference (RT: incongruent – congruent) on uninformed trials plotted as a function of dopamine synthesis capacity in the left dorsal caudate nucleus (ROI specified according to Mawlawi *et al.* and used by Aarts *et al.*). Shaded area around the regression line represents 95% confidence interval. RT (ms) = response time in milliseconds; K_i_ = [^18^F]DOPA uptake, reflecting dopamine synthesis capacity; *N* = 44.


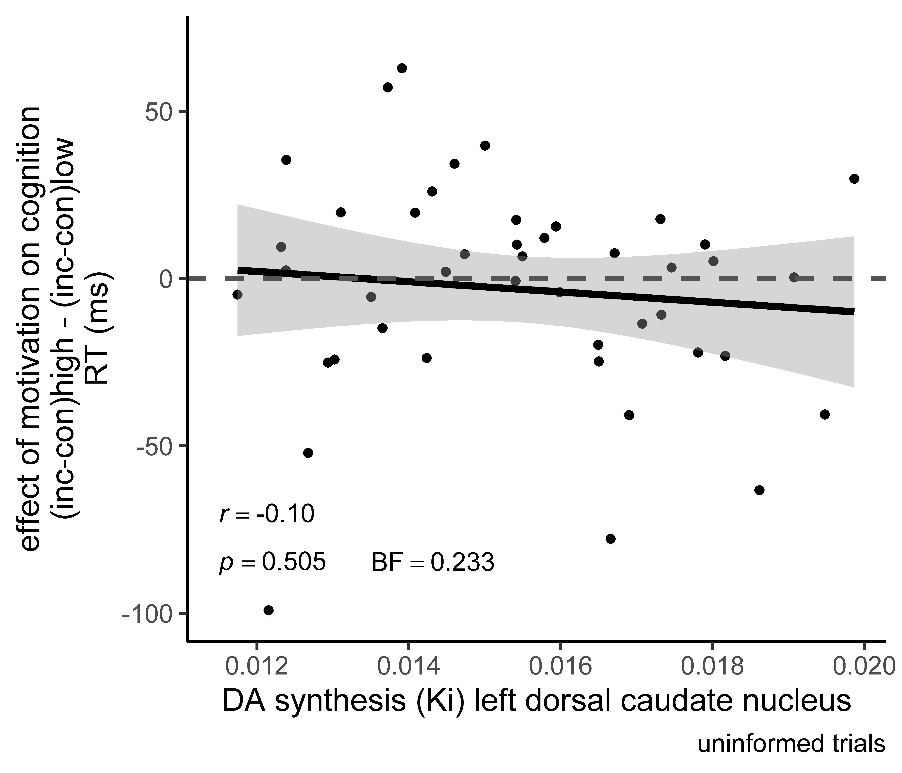


*detrimental*

**No evidence for a quadratic relationship between dopamine synthesis capacity and Stroop interference on low reward trials**

Our primary hypothesis was that dopamine synthesis capacity would be associated linearly with the effect of reward on Stroop performance. This hypothesis was based on a putative inverted-U shaped relationship between dopamine and Stroop performance, whereby further increases in dopamine elicited by the promise of reward would shift dopamine levels from suboptimal to optimal in low-dopamine participants, but from optimal to supra-optimal in high-dopamine participants. In addition to testing the linear effects of reward, we also explored quadratic effects under low-reward (putative baseline) conditions. To this end, we ran linear regressions (separately for each ROI) in R using the lm function, including the quadratic z-scored K_i_ term as independent variable and Stroop interference on the uninformed low reward trials as dependent variable. A *p*-value below a Bonferroni-corrected alpha-level of 0.0083 (0.05÷6 ROIs) was considered significant. We additionally ran the same Bayesian linear regressions in JASP to obtain a Bayes Factor for the effect. Results are displayed in Fig. S3. Although this revealed that Stroop interference was indeed strongest for individuals with the lowest and highest dopamine synthesis capacity in the right caudate nucleus (*R*^2^ = 0.159, *p* = 0.007, BF = 2.99), this relationship was driven by the participant with the highest Stroop interference score, who was an outlier according to a Grubbs’ test (*G* = 4.0, *p* = 1.6e^-4^). Without this participant the quadratic relationship was not present anymore (*R*^2^ = 0.03, *p* = 0.292, BF = 0.481). There were no significant quadratic relationships between dopamine synthesis capacity in the other ROIs and Stroop interference. Crucially, the quadratic effect between dopamine synthesis capacity (in the left dorsal caudate nucleus, as specified according to Mawlawi et al. ^3^ and Stroop interference was also not present in the study by Aarts and colleagues (Fig. S4).


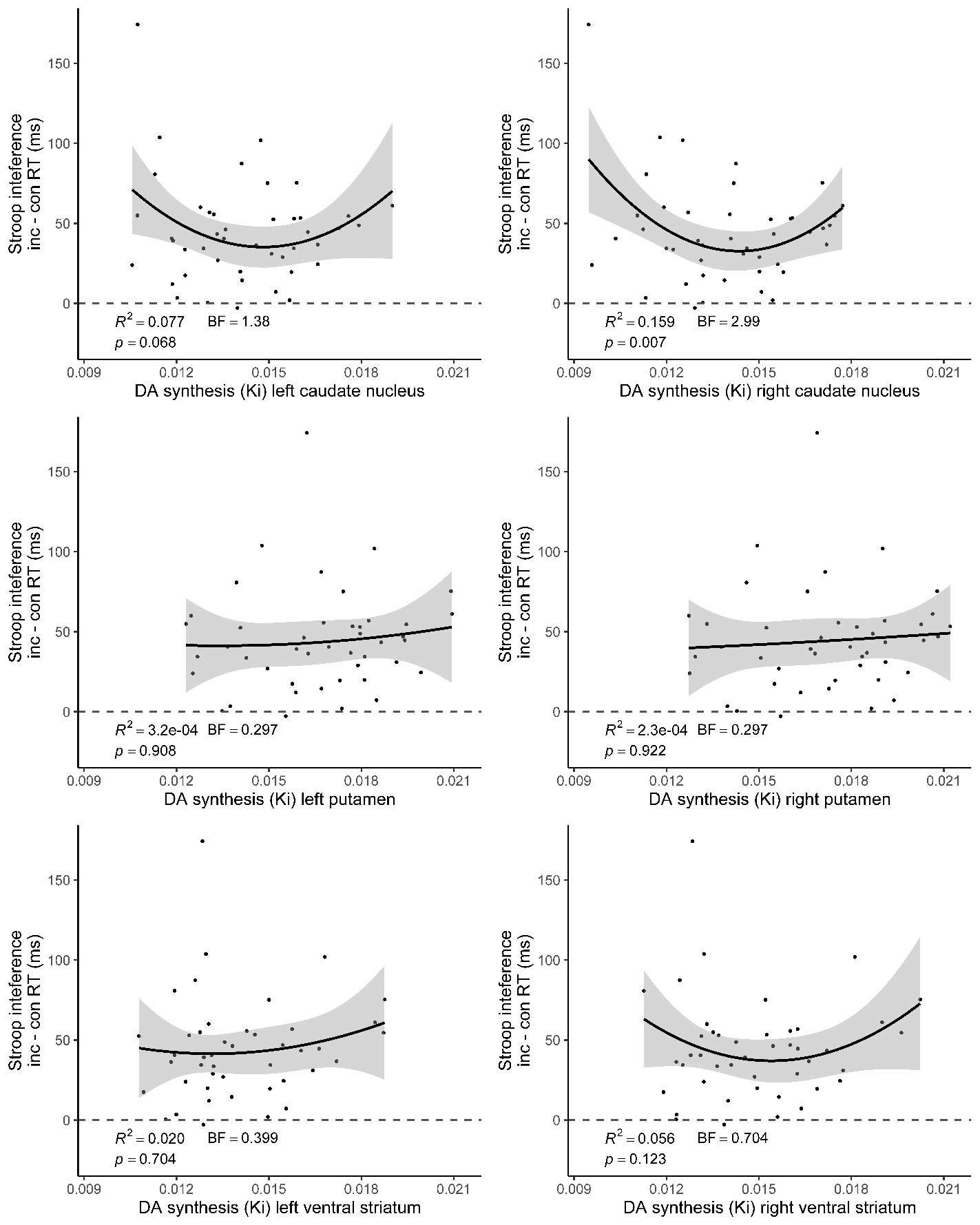
**Figure S3.** Quadratic K_i_-related effect on Stroop interference (mean RT incongruent trials minus mean RT congruent trials) in the six ROIs for uninformed low reward trials. Shaded area around the regression line represents 95% confidence interval. RT (ms) = response time in milliseconds; K_i_ = [^18^F]DOPA uptake, reflecting dopamine synthesis capacity; *N* = 44. NB: Quadratic relationship between dopamine synthesis capacity in the right caudate nucleus and Stroop interference without the participants with the highest Stroop interference: *R*^2^ = 0.03, *p* = 0.292, BF = 0.481.


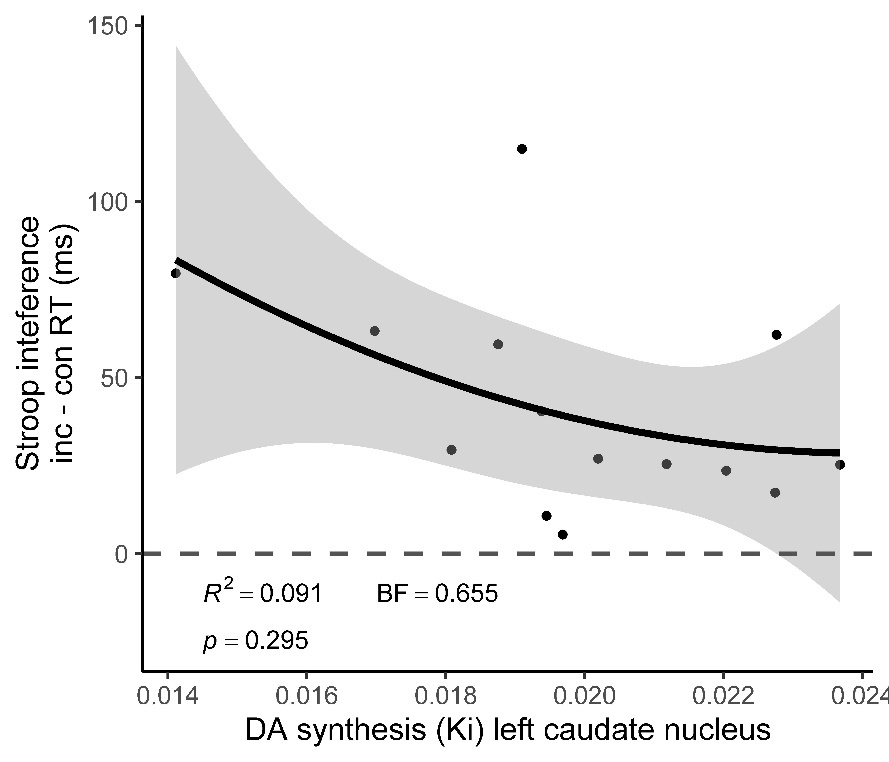
**Figure S4.** Quadratic K_i_-related effect on Stroop interference (mean RT incongruent trials minus mean RT congruent trials) in the left dorsal caudate nucleus for uninformed low reward trials in Aarts *et al*. Shaded area around the regression line represents 95% confidence interval. RT (ms) = response time in milliseconds; K_i_ = [^18^F]DOPA uptake, reflecting dopamine synthesis capacity; *N* = 14.

Both in the current sample (Table S1) and the original sample (Table S2), baseline dopamine synthesis capacity was not associated with response times, neither in interaction with reward nor as main effect:

| **Table S1**. Effect of dopamine synthesis capacity and dopamine synthesis capacity x reward on response times in the current sample. Separate rmANOVA for each of the six regions of interest, including reward, congruency and information as within-subjects factors and dopamine synthesis capacity as covariate. N = 44. | | | | |  |
| --- | --- | --- | --- | --- | --- |
|  | **DAsynth** | | **Reward x DAsynth** | | |
|  | *F*_(1,42)_ | *p* | *F*_(1,42)_ | *p* | |
| Left caudate nucleus | 2.4 | 0.128 | 3.1 | 0.084 | |
| Right caudate nucleus^1^ | 7.5 | 0.009 | 3.8 | 0.058 | |
| Left putamen | 0.0 | 0.833 | 1.1 | 0.299 | |
| Right putamen | 0.1 | 0.804 | 1.3 | 0.268 | |
| Left ventral striatum | 0.1 | 0.771 | 1.3 | 0.261 | |
| Right ventral striatum | 0.3 | 0.596 | 2.9 | 0.095 | |
| ^1^Corresponds to a negative correlation between dopamine synthesis capacity and response times. Effects when 1 participant with low dopamine synthesis capacity in the right caudate nucleus and an average RT of 4 standard deviations above the group mean was excluded: DAsynth: *F*_(1,41)_ = 3.0, *p* = 0.092; reward x DAsynth: *F*_(1,41)_ = 0.4, *p* = 0.535. | | | | | |

| **Table S2**. Effect of dopamine synthesis capacity and dopamine synthesis capacity x reward on response times in Aarts *et al*. Separate rmANOVA for each of the six regions of interest, including reward, congruency and information as within-subjects factors and dopamine synthesis capacity as covariate. N = 14. | | | | |
| --- | --- | --- | --- | --- |
|  | **DAsynth** | | **Reward x DAsynth** | |
|  | *F*_(1,12)_ | *p* | *F*_(1,12)_ | *p* |
| Left caudate nucleus | 0.1 | 0.738 | 0.0 | 0.930 |
| Right caudate nucleus | 0.0 | 0.855 | 0.0 | 0.871 |
| Left putamen | 0.0 | 0.966 | 0.0 | 0.965 |
| Right putamen | 0.0 | 0.907 | 0.1 | 0.817 |
| Left ventral striatum | 0.2 | 0.702 | 0.0 | 0.893 |
| Right ventral striatum | 0.1 | 0.780 | 0.3 | 0.567 |

**No evidence for a quadratic relationship between dopamine synthesis capacity and the effect of motivation on Stroop interference on uninformed trials**

Although our hypothesis concerned a linear relationship between dopamine synthesis capacity and the effect of motivation on Stroop interference, a visual inspection of Fig. 2 led us to explore a quadratic relationship for the left and right caudate nucleus. To this end, we ran linear regressions (separately for both ROIs) in R using the lm function, including the quadratic z-scored K_i_ term as independent variable and the effect of motivation on Stroop interference in terms of RT on the uninformed trials as dependent variable. We applied a strict alpha level of 0.0036 (0.05÷14; 12 linear relationships and 2 additional quadratic relationships) to account for these additional analyses assessing the relationship between dopamine synthesis capacity and the effect of motivation on Stroop interference. We additionally ran the same Bayesian linear regressions in JASP to obtain a Bayes Factor for the effect.

However, there was no significant quadratic relationships between the effect of motivation on Stroop interference and dopamine synthesis capacity in the left (*R^2^* = 0.05, *p* = 0.144, BF = 0.719) or the right caudate nucleus (*R^2^* = 0.11, *p* = 0.028, BF = 2.271).

| **Table S3**. Interaction effects in terms of response times (RT) and error rates obtained from the rmANOVAs with dopamine synthesis capacity in each ROI as a single covariate. The dependent variable is Stroop interference (mean RT or error rate on incongruent trials minus mean RT or error rate on congruent trials). The sample is matched to the original sample from Aarts *et al.* in terms of age, resulting in N = 26. | | | | | | |
| --- | --- | --- | --- | --- | --- | --- |
|  | **Reward x information x DAsynth** | | | **Reward x DAsynth** | | |
|  | *F*_(1,24)_ | *p* | BF_INC_ | *F*_(1,24)_ | *p* | BF_INC_ |
| RT |  |  |  |  |  |  |
| Left caudate nucleus | 0.0 | 0.991 | 0.012 | 0.9 | 0.345 | 0.081 |
| Right caudate nucleus | 0.1 | 0.738 | 0.008 | 1.5 | 0.240 | 0.078 |
| Left putamen | 0.2 | 0.655 | 0.020 | 0.1 | 0.749 | 0.084 |
| Right putamen | 0.1 | 0.710 | 0.017 | 0.1 | 0.707 | 0.076 |
| Left ventral striatum | 0.0 | 0.931 | 0.015 | 0.1 | 0.737 | 0.070 |
| Right ventral striatum | 0.1 | 0.797 | 0.033 | 0.0 | 0.963 | 0.093 |
| Error rate |  |  |  |  |  |  |
| Left caudate nucleus | 0.1 | 0.806 | 0.121 | 0.1 | 0.717 | 0.231 |
| Right caudate nucleus | 0.2 | 0.683 | 0.180 | 0.0 | 0.848 | 0.281 |
| Left putamen | 1.5 | 0.236 | 0.351 | 0.0 | 0.879 | 0.369 |
| Right putamen | 2.4 | 0.138 | 0.391 | 0.3 | 0.583 | 0.432 |
| Left ventral striatum | 0.7 | 0.397 | 0.138 | 0.0 | 0.933 | 0.238 |
| Right ventral striatum | 0.2 | 0.674 | 0.218 | 0.0 | 0.931 | 0.323 |
| Note: *p*-values below a Bonferroni-corrected alpha-value of 0.0042 were considered significant. | | | | | | |

| **Table S4**. Interaction effects in terms of response times (RT) and error rates obtained from the rmANOVAs with dopamine synthesis capacity in each ROI as a single covariate. The dependent variable is Stroop interference (mean RT or error rate on incongruent trials minus mean RT or error rate on congruent trials). The sample is matched to the original sample from Aarts *et al.* in terms of individual average RT across all trials, resulting in N = 29. | | | | | | |
| --- | --- | --- | --- | --- | --- | --- |
|  | **Reward x information x DAsynth** | | | **Reward x DAsynth** | | |
|  | *F*_(1,27)_ | *p* | BF_INC_ | *F*_(1,27)_ | *p* | BF_INC_ |
| RT |  |  |  |  |  |  |
| Left caudate nucleus | 0.5 | 0.465 | 0.094 | 1.2 | 0.282 | 0.099 |
| Right caudate nucleus | 0.1 | 0.780 | 0.009 | 2.5 | 0.125 | 0.098 |
| Left putamen | 2.2 | 0.153 | 0.005 | 0.0 | 0.938 | 0.037 |
| Right putamen | 2.2 | 0.147 | 0.005 | 0.0 | 0.932 | 0.037 |
| Left ventral striatum | 0.6 | 0.459 | 0.004 | 0.0 | 0.935 | 0.039 |
| Right ventral striatum | 0.2 | 0.636 | 0.004 | 0.1 | 0.730 | 0.048 |
| Error rate |  |  |  |  |  |  |
| Left caudate nucleus | 0.2 | 0.651 | 0.016 | 0.0 | 0.884 | 0.041 |
| Right caudate nucleus | 0.0 | 0.884 | 0.014 | 0.0 | 0.945 | 0.054 |
| Left putamen | 2.8 | 0.106 | 0.028 | 0.2 | 0.697 | 0.082 |
| Right putamen | 5.6 | 0.025^1^ | 0.041 | 0.0 | 0.936 | 0.072 |
| Left ventral striatum | 0.4 | 0.551 | 0.013 | 0.5 | 0.490 | 0.073 |
| Right ventral striatum | 0.4 | 0.517 | 0.014 | 0.0 | 0.954 | 0.085 |
| Note: *p*-values below a Bonferroni-corrected alpha-value of 0.0042 were considered significant.  ^1^*p*-value does not survive correction for multiple comparisons. However, for clarity we report the interaction effect of reward x DAsynth in the right Putamen on Stroop interference (error rate): informed trials: *r* = -0.21, *p* = 0.266; uninformed trials: *r* = 0.29, *p* = 0.131. | | | | | | |

| **Table S5**. Interaction effects obtained from multiple linear regression analyses assessing the effect of individual average RT across all trials on motivational effects on Stroop interference (incongruent trials minus congruent trials) in terms of response times (RT) and error rates. Separate analysis for each ROI. | | | | | | | | |
| --- | --- | --- | --- | --- | --- | --- | --- | --- |
|  | **Reward x information x DAsynth** | | **Reward x**  **DAsynth** | | **Reward x information x DAsynth x RT** | | **Reward x**  **DAsynth x RT** | |
|  | β | *p* | β | *p* | β | *p* | β | *p* |
| RT |  |  |  |  |  |  |  |  |
| Left caudate nucleus | 5.2e^3^ | 0.207 | -7.3e^3^ | 0.260 | -5.9e^1^ | 0.416 | 1.3e^2^ | 0.251 |
| Right caudate nucleus | 4.7e^3^ | 0.260 | -6.3e^3^ | 0.330 | -4.2e^1^ | 0.510 | 1.1e^2^ | 0.280 |
| Left putamen | 4.4e^3^ | 0.248 | -7.0e^3^ | 0.249 | 1.8e^1^ | 0.870 | 1.3e^1^ | 0.939 |
| Right putamen | 4.5e^3^ | 0.219 | -7.1e^3^ | 0.223 | -2.7e^1^ | 0.797 | 8.8e^1^ | 0.602 |
| Left ventral striatum | 3.8e^3^ | 0.371 | -6.5e^3^ | 0.334 | -2.1e^1^ | 0.819 | 7.2e^1^ | 0.819 |
| Right ventral striatum | 3.1e^3^ | 0.424 | -5.1e^3^ | 0.405 | -5.1e^1^ | 0.486 | 1.1e^2^ | 0.333 |
| Error rate |  |  |  |  |  |  |  |  |
| Left caudate nucleus | 3.0 | 0.878 | 2.5 | 0.934 | 1.1e^-1^ | 0.738 | 6.3e^-2^ | 0.906 |
| Right caudate nucleus | -3.8 | 0.846 | 1.2e^1^ | 0.699 | 2.4e^-1^ | 0.419 | -1.3e^-1^ | 0.780 |
| Left putamen | -8.2 | 0.648 | 1.4e^1^ | 0.622 | -2.2e^-1^ | 0.661 | 5.4e^-1^ | 0.501 |
| Right putamen | -1.2e^1^ | 0.505 | 2.2e^1^ | 0.416 | -1.8e^-1^ | 0.717 | 4.9e^-1^ | 0.535 |
| Left ventral striatum | -3.8e^-2^ | 0.998 | -2.1 | 0.947 | 1.0e^-1^ | 0.814 | 1.0e^-1^ | 0.881 |
| Right ventral striatum | 3.8 | 0.834 | -5.6 | 0.847 | 2.1e^-3^ | 0.995 | 2.5e^-1^ | 0.650 |
| Model: stroop_effect ~ DAsynth x reward x information x average RT | | | | | | | | |

**REFERENCES**

1. Aarts, E. *et al.* Dopamine and the Cognitive Downside of a Promised Bonus. *Psychol. Sci.* **25**, 1003–1009 (2014).

2. Piray, P., Ouden, H. E. M. Den, Schaaf, M. E. Van Der, Toni, I. & Cools, R. Dopaminergic Modulation of the Functional Ventrodorsal Architecture of the Human Striatum. *Cereb. Cortex* **27**, 485–495 (2017).

3. Mawlawi, O. *et al.* Imaging human mesolimbic dopamine transmission with positron emission tomography: I. Accuracy and precision of D2 receptor parameter measurements in ventral striatum. *J. Cereb. Blood Flow Metab.* **21**, 1034–1057 (2001).
